# Dysphagia Prevalence in Brazil, UK, China, and Indonesia and Dysphagic Patient Preferences

**DOI:** 10.3390/healthcare12181827

**Published:** 2024-09-12

**Authors:** Joseph Cook, Molly Sapia, Chris Walker, Melissa Pittaoulis

**Affiliations:** 1 Viatris, Canonsburg, PA 15317, USA; chris.walker@viatris.com; 2 NERA Economic Consulting, New York, NY 10036, USA; molly.sapia@nera.com (M.S.); melissa.pittaoulis@nera.com (M.P.)

**Keywords:** difficulty swallowing, dysphagia, epidemiology, patient adherence, diagnosis, patient preference

## Abstract

Background: Dysphagia is common, but there is limited information about its prevalence and patient preferences regarding dosage forms (oral solids, liquids, topical, etc.) in Brazil, China, the United Kingdom (UK), and Indonesia. Methods: We conducted an online survey of 1000 adults from each country, without any required disease, to estimate the prevalence of dysphagia in these four nations and the dosage form preferences among UK patients. Results: A total of 36.9%, 40.5%, 54.9%, and 64.5% from the UK, Indonesia, Brazil, and China, respectively, had an Eating Assessment Tool (EAT-10) score of ≥3 (indicative of dysphagia). Only 2% of UK respondents and 5% of Brazilian respondents reported a formal diagnosis of dysphagia. Indonesian (74%) and Chinese respondents (77%) were more likely than Brazilian (52%) and UK respondents (45%) to report that their swallowing problems affected their ability to adhere to medication instructions. Liquids were the oral medication formulation most preferred by those who reported difficulty swallowing. Conclusions: To conclude, substantial populations have difficulty swallowing, which can translate into an access issue for medical treatment. The availability of people’s preferred dosage forms may help alleviate the adherence issues associated with difficulty swallowing and the concomitant effects on health outcomes.

## 1. Introduction

Dysphagia, or difficulty swallowing, can affect people of any age, but it is common in the elderly [1]. Studies estimating the prevalence of dysphagia generally focus on at-risk or elderly populations. For example, researchers studying dysphagia in the US found that 33% of their elderly participants noted a current swallowing problem [2]. In a survey of nearly 6000 members of the 65 and older population in China, researchers identified that 39.4% of the respondents had dysphagia [3]. It is important to understand how common it is for people to have trouble swallowing medications, and what forms of medication that population prefers. Non-adherence to treatment plans can lead to poorer outcomes. Medications in the form of liquids or injections, for example, may be preferable for people with dysphagia [4]. To foster adherence, patients must be given a more active role in their treatment process and their preferences must be considered [5]. To investigate the prevalence of dysphagia and difficulty swallowing more generally, we conducted an online survey of the adult populations of four countries: the United Kingdom (UK), Brazil, China, and Indonesia. Respondents in the UK were also asked questions to ascertain their preferences for particular dosage forms of medication. The rationale for this study was to better inform physician prescription choice and pharmaceutical drug development, and our research objective was to better understand how common difficulty swallowing might be in these countries, and which dosage forms people with difficulty swallowing prefer. These preferred dosage forms may help to increase adherence and improve health outcomes.

## 2. Methods

Here, we utilize the EAT-10 instrument. The EAT-10 is a quickly administered and easily scored dysphagia assessment that can be used to obtain an overview of symptom severity, quality of life, and treatment efficacy [6]. It is used around the world and is especially well-equipped to detect swallowing disorders in otherwise healthy populations. In a meta-analysis evaluating the accuracy of the EAT-10, Zhang et al. concluded that it has strong internal consistency and good diagnostic performance [7]. Respondents to our survey were shown the scenarios of the EAT-10 tool and asked to rate each of those ten on a scale of 0 (no problem) to 4 (severe problem). We also asked five questions specifically about difficulty swallowing medications. Finally, we also asked respondents if they had ever discussed swallowing issues with their healthcare providers or been diagnosed with dysphagia or odynophagia. Patients with difficulty taking their medications are more likely to have problems with adherence. By looking at the general population, rather than a particular patient population, our approach allows us to confirm the results of previous research showing that not all difficulty swallowing is identified [8], as well as assess the prevalence of dysphagia in the general population.

Our survey also included an additional module for the UK respondents in which we asked about their experiences and preferences for taking different types of medication, as well as any coping mechanisms respondents have tried for making it easier to swallow medications.

The survey was conducted online between October and November 2021 and included 4000 adults in the UK, Brazil, China, and Indonesia. This set of countries provides a mix geographically and culturally, although other selections would as well. The online survey was programmed and hosted by Dynata, the largest-reaching market research firm in the world, which has a reach of 62 million people worldwide [9]. Dynata partnered with the global translation firm Cetra to translate the entire survey from American English to Portuguese, simplified Chinese, Indonesian and UK English localization. The survey was non-interventional and anonymized and, therefore, did not require ethics review; however, Dynata’s panel management is compliant with all privacy laws and has received top-tier scores in data privacy when audited by a third-party organization [10]. When panelists sign up to be a part of Dynata’s survey panel, they consent to data collection and the processing of said data. Dynata’s panelists are also able to review Dynata’s privacy policy before participating in any surveys and are allowed to withdraw consent at any time [11]. The authors did not have access to any respondents’ personally identifiable data. The questionnaire is available as a Supplementary Materials.

Respondents qualified for participation by being 18 or older, living in either Brazil, China, Indonesia, or the UK, and passing quality control measures, including passing a Google reCAPTCHA question, and selecting the number four from a list of numbers. These measures helped to ensure that the respondent was a human being who was paying attention, and not a robot. Further, if respondents entered an age or gender that differed from what Dynata had in its records, they were excluded from participation.

Data were analyzed using the statistical software package Stata 17 (Stata Corp. LLC, College Station, TX, USA). The statistical significance for comparing differences by country, or differences in the results between patient groups (e.g., with and without an indication of dysphagia), where applicable, was determined using Mann–Whitney tests, two-tailed *t*-tests with a *p*-value of *p* < 0.05, or, in one instance, a logistic regression for country differences. Questions based on the EAT-10 were analyzed in accordance with the traditional procedure for determining whether the results suggest the presence of dysphagia. EAT-10 scores were calculated by totaling the numbers the respondent selected for all ten items (ranging 0 to 4), and thus the total possible score per respondent ranged from 0 to 40. Consistent with other literature, we consider a total score of 3 or higher indicative of the presence of dysphagia [6,7].

### Description of the Sample

The age and sex distributions for all respondents across the four countries are shown in Table 1. 

As shown, the sample is split rather evenly on gender, with a wide range of ages. However, there are some differences. Brazil has the fewest males, and the Mann–Whitney test found that it has a gender distribution statistically different from the other three countries: Brazil and China (*p* = 0.0032), Brazil and Indonesia (*p =* 0.000), and the UK and Brazil (*p* = 0.0396). Indonesia has the most males, and the Mann–Whitney test found that its gender distribution is statistically different only from the UK (*p* = 0.0032). Similarly, the UK population has the fewest participants in the youngest age group, and the Mann–Whitney test results confirm that the UK age distribution is statistically different from each of the other three countries: UK and Brazil (*p* = 0.0000), UK and China (*p* = 0.0000), and UK and Indonesia (*p* = 0.0000). We use these data as control variables later in the regression analysis. In Table 2, we present the share of respondents in each country who reported having any of a variety of prior health conditions that may correspond with dysphagia. These were not inclusion criteria, but merely those reported by the participants of the survey. While many patients reported having one or more of these comorbidities commonly linked with dysphagia, a substantial portion did not. Past research has shown that the populations with the highest risk of developing dysphagia are those with neurodegenerative diseases such as Parkinson’s disease, dementia, head and neck cancer (HNC), children with cleft lips and palates, and those recovering from stroke [3,12,13,14,15].

## 3. Results

### 3.1. Dysphagia Prevalence

As noted above, the EAT-10 contains 10 scenarios, and respondents were asked to rate each scenario according to how problematic it is for them, with 0 meaning “no problem” and 4 meaning it is a “severe problem”. The mean score for each individual scenario from 0 to 4 within each country is presented in Table 3.

Once the ten scores are summed together, EAT-10 scores can range from 0 to 40, and experts consider a total score of 3 or higher to indicate the presence of dysphagia. The mean EAT-10 score was 5.3 among UK respondents, 5.6 among Brazilian respondents, 7.8 among Indonesian respondents, and 10.0 among Chinese respondents. As shown in Table 4, the percentage of respondents with EAT-10 scores of ≥3 was 36.9% in the United Kingdom, 40.5% in Brazil, 54.9% in Indonesia, and 64.5% in China. Indonesian and Chinese respondents more frequently gave answers indicative of dysphagia than respondents from the UK and Brazil. For each country, the item rated as a problem (score > 0) by the highest percentage of respondents was item No. 5: “Swallowing pills takes extra effort” (UK = 44%, Brazil = 49%, Indonesia = 50%, and China = 57%).

In our survey, aside from the EAT-10, respondents were also asked explicitly if they have trouble swallowing medications (see Table 4). The share of respondents reporting difficulty at this question ranged from one-quarter to just over 40% across the four countries: Indonesia (25.0%), the UK (32.3%), Brazil (38.2%), and China (43.6%).

We also asked if respondents had ever been diagnosed by a healthcare provider with a swallowing condition. Those results ranged from 7% in the UK to 38% in China. In Table 4, we present a summary of the reported prevalence of dysphagic tendencies across these three measures. As shown, using the EAT-10 results in higher estimates of prevalence than asking respondents if they have difficulty swallowing medications or if they have been diagnosed with a swallowing condition by a health care provider.

### 3.2. Prescription Adherence

As discussed, substantial shares of the general population in the UK, Indonesia, Brazil, and China report difficulty swallowing, and especially difficulty swallowing pills. Understanding the prevalence of dysphagia is important in considering how respondents adhere to medication treatments prescribed by their physicians. We asked those who said they had trouble swallowing medications various follow-up questions about how, if at all, that affects their ability to adhere to prescription instructions. As shown in Table 5, we found that over half of those with difficulty swallowing medications indicated that that difficulty occurred either every time they take medication or most times they take medication in the UK (56%); China (55%); Brazil (66%); and Indonesia (62%). Further, many respondents reported that their difficulty swallowing affected their ability to adhere to medication instructions: in the Indonesian (74%), China (77%), Brazil (52%) and UK respondents (45%), an average of 61.7% selected yes to this question across the four countries. Looking at all respondents in these countries, not just those with swallowing difficulties, the percentage of the general population who reported having trouble adhering to their medication instructions because of swallowing difficulties ranged from 14% (UK) to 33% (China).

We also asked respondents who have trouble swallowing medications whether they had engaged in any of five behaviors related to their prescriptions because of their difficulty swallowing, the results of which are reported in Table 5. The majority of respondents in each country who had reported difficulty swallowing medication indicated they had done at least one of the following: (1) taken fewer pills or tablets at a time than recommended; (2) taken medication less frequently than the recommended schedule; (3) sought alternative treatments that do not include medication; (4) quit taking a medication; and (5) not filled a prescription at all. Among all respondents, not just those who have difficulty swallowing medication, the percentage of respondents who reported one of these five behaviors was 22% in the UK and Indonesia, 31% in Brazil, and 38% in China. An average of 81% of the respondents who reported difficulty swallowing medications had engaged in at least one of these behaviors (ranging from 66.9% in UK to 89.6% in Indonesia). Looking at the entire populations and not only those with difficulty swallowing medications, an average of 28.4% of respondents had engaged in one of these five nonadherence behaviors, ranging from 21.6% in the UK to 38.1% in China.

### 3.3. Patient Preferences

As shown, in all four countries surveyed, large shares of the general population have dysphagic tendencies, and many of those appear to engage in prescription nonadherence because of their difficulty swallowing. Respondents in the UK were asked additional questions about their experiences taking medication and their preferences for certain formulations. 

We observed that, by far, the most common form of medication taken by these respondents is oral medication, with 73% of respondents indicating they have taken this form of medication in their adult life. Focusing on oral medication, respondents with experience taking this type of medication were more likely to have experience with capsules (80%) and pills/tablets (79%) than liquids (59%) or tablets that dissolve in your mouth (50%). Prior work on the relative difficulty of swallowing different dosage formulations has illustrated similar rankings. [16] 

We asked the respondents about their preferences for different medication formulations. Respondents were presented with 10 different medication formulations and asked to imagine that they had the choice to select the form in which their medication was administered. Respondents were instructed to allocate 100 points across the 10 formulations, giving more points to those formulations they preferred more and less points to those they did not prefer. Respondents were permitted to allocate zero points to a formulation if they did not have any preference for that formulation. In Table 6, we present the results for this question for different groups. Looking at all respondents, pills or tablets received the highest average point allocation at 29.4 points. This was followed by capsules (20.4) and liquids (15.3). Next, we compared the average number of points allocated for each medicinal formulation for those who have no difficulty swallowing to those who do report difficulty swallowing, either by saying yes to having difficulty swallowing medications, and/or having a score of 3 or more on the EAT-10. Respondents who have difficulty swallowing allocated, on average, double the points to liquid formulations (20.8) compared to those with no difficulty swallowing (10.2, two-tailed *t*-test *p* = 0.000). Looking at tablets that dissolve in the mouth, those who have difficulty swallowing were more likely than those with no difficulty swallowing to prefer this form, i.e., allocate more points to this form, (10.9 compared to 6.4, two-tailed *t*-test *p* = 0.000). Compared to respondents who have difficulty swallowing, respondents with no difficulty swallowing had greater preferences for pills or tablets that must be swallowed (37.8 compared to 20.1, two-tailed *t*-test *p* = 0.000) and capsules (24.9 compared to 15.5, two-tailed *t*-test *p* = 0.000).

Next, we compare formulation preferences across three groups: in those with no difficulty swallowing, those with swallowing difficulties but no diagnosis, and those with a diagnosed swallowing condition, we see that being diagnosed with dysphagia is mostly unrelated to formulation preferences (Table 7). Those with no difficulty swallowing have a higher preference for capsules and pills or tablets that need swallowing than other formulations, and their preferences for these forms are higher than those of respondents with difficulty swallowing but no diagnosis and those diagnosed with a swallowing condition (*p* = 0.000 for each of these four two-tailed *t*-tests). Respondents with swallowing problems but no diagnosis and those not diagnosed with dysphagia expressed similar preferences for all but one formulation. Those without a diagnosis tended to have stronger preferences for liquids than those with a diagnosis. Both groups of respondents with difficulty swallowing (undiagnosed and diagnosed) had a greater preference for tablets that dissolve in your mouth (averaging at about 11) compared to those with no swallowing difficulties (at about a 6, *p* = 0.000 for both *t*-tests).

## 4. Discussion

In this study, we measured the prevalence of swallowing problems among the general adult population in four countries using self-reported survey data. The first measure applied was the EAT-10 tool used widely around the world to identify cases of suspected dysphagia. Scores ≥ 3 are typically cause for patient referral. The current study found that administering the EAT-10 tool to a general population of adults resulted in reasonably high estimates of suspected dysphagia in all four countries, but that China (65%) and Indonesia (55%) had higher reports of swallowing problems than the UK (37%) and Brazil (41%) (all four two-tailed *t*-tests have a *p*-value of 0.000). Smaller percentages of respondents in the UK (4%), Brazil (7%), and Indonesia (9%) reported having been diagnosed with these conditions. In contrast, in China, 38% of respondents reported having been diagnosed with a swallowing condition. We obtained similar results with a logistic regression of self-reported diagnosis on age, gender, EAT-10 score, and country. We used the UK as the reference, and the estimated odds ratios and *p*-values for the country odds ratios were as follows: Brazil 1.389 (*p* = 0.061), China 6.514 (*p* = 0.000) and Indonesia 1.633 (*p* = 0.003). Age and the EAT-10 score were also statistically significant at the 0.05 level, with 0.824 (*p* = 0.000) and 1.108 (*p* = 0.000), respectively; however, the categorical variable of male (female as the reference) was not, at 0.940 (*p* = 0.528). Age has a positive relationship with dysphagia diagnosis; older participants are more likely to report a dysphagia diagnosis. In sum, although there are differences in the distributions of age and gender across the country samples, when controlling for these differences, we still see differences across the countries, as shown in Table 1. See Table 8. The higher prevalence of swallowing problems in China is an area for further research. Cultural or environmental factors may lead to higher reports of swallowing difficulties. High levels of pollution are known to be associated with adverse health problems, such as asthma and breathing difficulties. It would be interesting to learn whether pollution, of which China has high levels, also contributes to swallowing problems. 

One of the objectives of this study was to measure the prevalence of swallowing problems in general, including in those who have not received a formal diagnosis. A substantial portion of the adult population in these four countries reported swallowing difficulties related to medication (Indonesia, 25%; UK, 32%; Brazil, 38%; China, 44%). For many of these respondents, these problems were frequent, occurring either most of the time or every time they take medication. Furthermore, respondents also reported that as a result of these problems, they sometimes have difficulty adhering to their medication schedules, which may lead to behaviors such as taking fewer pills or tablets than prescribed or taking their medication less frequently than scheduled. 

Poor medication adherence in patients can result in negative outcomes including a worsening of disease. In a systematic review of adherence studies of diagnosed dysphagia patients, the authors found that adherence rates ranged from 22% to 52% [5]. 

The survey found that there may be a disconnect between the types of medication that the respondents took and the formulations that they prefer. For example, although there is a high preference for liquids, only 20% of respondents who currently take medication report that their medication is in this form. The preferences of those who have a diagnosis are largely similar to the preferences of those who report difficulty swallowing but have not been diagnosed, and these preferences are different from those who have no difficulty swallowing. This suggests that, when examining people’s preferences as patients, the presence of a swallowing problem might be a more important consideration than a formal diagnosis. Such results highlight the importance of conducting research among the general population and not just those who have formal diagnoses when examining patient preferences. By focusing this survey on preferences around dosage forms, we addressed the broader need to align medication dosage forms and methods of delivery with patient needs, thus helping foster better access and outcomes. Surveying people about how they, as patients, would prefer their medications to be administered may foster better patient outcomes.

Our survey was carried out in multiple countries using a validated tool (EAT-10), as well as using additional direct questions on difficulty swallowing and diagnoses. Further research could look at additional countries, which might have additional insights to share on pollution and other potential factors influencing difficulty swallowing and ways to address it. This is particularly true with respect to our preference results around dosage forms and methods of delivery, which were limited to the UK, where additional precision could also be sought. 

### Limitations

While providing international data on different approaches to measuring people’s difficulty swallowing medications and offering a new comparison between the preferences of people with and without swallowing difficulties, this study has some limitations. While we used the EAT-10 to measure dysphagia, there are other instruments that could be used. In the future, researchers might use additional instruments or expand the survey to include more or other countries, either for prevalence or preferences. Given the online form of the survey, the dysphagia diagnoses are not clinically validated, and the data might have benefited from that; however, we tried to compensate by using multiple indicators of difficulty swallowing. Lastly, while we measure patient preferences for different drug forms, we cannot here evaluate the safety of each form for those with difficulty swallowing. In other words, there may be a difference between the form that patients prefer and the form that is actually safe for them to swallow.

Further, surveys can be subject to bias, like recall bias (remembering things incorrectly) or response bias (providing inaccurate answers). To reduce recall bias, we asked the respondents about the swallowing difficulties they were currently experiencing, limiting the need to call upon experience. It is possible that recall bias affected respondents’ ability to remember the diagnoses they received; however, if a diagnosis were forgotten due to the passage of time, we might expect underreporting, which could imply our results understate the health issue. To reduce response bias, we asked respondents non-leading questions and avoided a loaded question structure. In the future, researchers may replicate this study to address selection bias.

## 5. Conclusions

This study demonstrates that substantial populations in the four countries sampled (Brazil, China, Indonesia, and the UK) have difficulty swallowing medicines, negatively impacting their adherence and health outcomes. Our UK results suggest that consistent with people’s preferences, liquid and orally disintegrating dosage forms may provide at least a partial solution. To conclude, substantial populations appear to have dysphagia or difficulty swallowing, which can translate into an access issue for medical treatment. The availability of preferred dosage forms, and particularly liquids, may help alleviate the adherence issues associated with dysphagia and the concomitant effects on health outcomes.

## Figures and Tables

**Table 1:** Respondents’ age and sex distribution by country.

	UK	China	Brazil	Indonesia
Males				
18–34	14.7%	21.5%	18.1%	21.7%
35–54	15.3%	19.5%	16.9%	24.0%
55+	20.5%	11.5%	10.9%	9.4%
Females				
18–34	12.0%	22.0%	25.0%	18.7%
35–54	18.5%	16.6%	20.2%	15.5%
55+	19.0%	8.9%	8.9%	10.7%
Total	100.0%	100.0%	100.0%	100.0%
Number of Respondents	1000	1000	1000	1000

**Table 2:** Dysphagia-related health conditions reported by respondents.

	United Kingdom	China	Brazil	Indonesia
ALS/Lou Gehrig’s disease	0.8%	0.7%	0.3%	0.8%
Arthritis	14.8%	14.6%	5.3%	10.5%
Cleft lip/palate	1.2%	0.9%	0.3%	0.7%
Communication disorder	1.6%	2.1%	1.2%	2.1%
Esophagitis	2.6%	7.7%	3.3%	0.9%
Fibromyalgia	3.5%	2.2%	2.8%	0.4%
Gastroesophageal reflux disease (GORD/GERD)	9.4%	6.0%	14.2%	6.7%
Lower back pain	27.9%	6.0%	26.8%	17.0%
Migraine	17.4%	21.4%	34.7%	39.3%
Multiple sclerosis	1.4%	1.4%	0.7%	0.6%
Myasthenia gravis	0.2%	0.7%	0.2%	0.2%
Neuropathy	2.4%	1.2%	0.5%	1.7%
Parkinson’s disease	0.9%	0.8%	0.3%	0.6%
Pneumonia	2.4%	4.6%	9.4%	5.6%
Stroke	1.9%	1.3%	0.7%	2.3%
Other chronic pain syndrome not listed	8.5%	6.5%	7.0%	3.0%
None of the above	44.9%	51.8%	34.2%	42.0%
Number of Respondents	1000	1000	1000	1000

**Table 3:** Mean Score given to each EAT-10 item by country.

	United Kingdom		China		Brazil		Indonesia	
	Mean	S.D.	Mean	S.D.	Mean	S.D.	Mean	S.D.
My swallowing problem has caused me to lose weight.	0.36	0.87	0.90	1.18	0.43	1.00	0.62	1.16
My swallowing interferes with my ability to go out for meals.	0.44	0.91	1.01	1.25	0.49	1.02	0.70	1.19
Swallowing liquids takes extra effort.	0.41	0.89	0.80	1.13	0.41	0.96	0.57	1.10
Swallowing solids takes extra effort.	0.57	1.04	1.04	1.22	0.62	1.11	1.01	1.29
Swallowing pills takes extra effort.	0.91	1.23	1.19	1.29	0.98	1.24	0.96	1.22
Swallowing is painful.	0.45	0.92	0.98	1.20	0.46	1.03	0.69	1.16
The pleasure of eating is affected by my swallowing.	0.50	0.99	0.96	1.21	0.56	1.07	0.90	1.24
When I swallow food sticks in my throat.	0.58	1.02	1.02	1.17	0.57	1.09	0.88	1.25
I cough when I eat.	0.56	1.00	1.07	1.18	0.59	1.09	0.81	1.25
Swallowing is stressful.	0.48	0.96	1.02	1.22	0.46	0.99	0.63	1.16
Number of Respondents	1000		1000		1000		1000	

**Table 4:** Dysphagic tendencies prevalence measures by country.

	United Kingdom	China	Brazil	Indonesia
Share with a score of 3 or more on the EAT-10 instrument (indicative of dysphagia)	36.9%	64.5%	40.5%	54.9%
Share who reported that they have difficulty swallowing medications (Q2)	32.3%	43.6%	38.2%	25.0%
Share who said they have been diagnosed by a healthcare provider with a swallowing condition (Q8)	7.3%	38.1%	10.6%	14.1%
Share—Any of the above	47.7%	70.1%	52.9%	59.4%
Number of Respondents	1000	1000	1000	1000

**Table 5:** Summary of responses regarding nonadherence behaviors because of difficulty swallowing.

	United Kingdom	China	Brazil	Indonesia
How often do you have problems taking medication as instructed because of swallowing difficulty? (Q3)				
Every time I take a medication	22.0%	16.3%	29.8%	27.6%
Most times I take medication, but not every time	34.4%	38.5%	36.1%	34.0%
Sometimes, but not most of the time	35.9%	40.1%	29.1%	34.8%
Seldom or rarely	7.1%	5.0%	4.7%	2.0%
Does your difficulty swallowing medication affect your ability to follow instructions for how often or how long you should take your medication? (Q5)				
Yes	44.6%	76.6%	52.1%	73.6%
Have you ever done any of the following due to your difficulty swallowing medication? (Q6)				
Take fewer pills or tablets at a time than recommended	35.0%	51.6%	45.3%	59.6%
Take medication less frequently than the recommended schedule	31.0%	40.8%	30.4%	40.8%
Seek alternative treatments that do not include medication	22.9%	40.4%	39.5%	41.6%
Quit taking a medication	18.3%	11.0%	22.8%	17.2%
Not fill prescription at all	10.2%	7.8%	8.1%	12.4%
Any of these five behaviors	66.9%	87.4%	82.2%	89.6%
Number of Respondents	323	436	382	250

Respondents were only asked these questions if they indicated at Q2 that they have trouble swallowing medications. Only a selection of the response options are presented here for a summary.

**Table 6:** Comparison of means for the allocation of preference points for each dosage form, by difficulty swallowing reported.

	All	No Difficulty Swallowing Reported	Difficulty Swallowing Reported	*t*-Test *p*-Value
Liquid	15.25	10.24	20.81	0.000
Pills or tablets in pressed powder form that must be swallowed	29.40	37.81	20.07	0.000
Capsules (active part of the medicine is contained inside a plastic shell)	20.41	24.86	15.47	0.000
Tablets that dissolve in your mouth	8.51	6.36	10.89	0.000
Topical medication (e.g., gel, cream, or ointment)	7.66	6.95	8.44	0.097
Nasal spray	5.07	4.49	5.71	0.124
Inhaler-type medication	4.87	3.46	6.44	0.000
Patch	3.88	2.80	5.07	0.000
Self-administered injection	2.28	1.16	3.53	0.000
Injection by a health-care provider	2.67	1.87	3.57	0.000
Number of Respondents	1000	526	474	

“Difficulty Swallowing” is defined as having said yes to having difficulty swallowing medications in Q2, and/or having a score of a 3 or more on the EAT-10. The *t*-tests performed are two-sampled, two-tailed tests with unequal variances.

**Table 7:** Comparison of means for the allocation of preference points for each dosage form by reported difficulty and diagnosis.

	All	No Difficulty Swallowing Reported	Difficulty Swallowing Reported, but Not Diagnosed with a Swallowing Condition	Diagnosed with a Swallowing Condition	*t*-Test *p*-Value
Liquid	15.25	10.26	21.78	14.86	0.000
Pills or tablets in pressed powder form that must be swallowed	29.40	37.76	20.06	21.18	0.000
Capsules (active part of the medicine is contained inside a plastic shell)	20.41	24.87	15.74	14.27	0.000
Tablets that dissolve in your mouth	8.51	6.36	10.73	11.62	0.000
Topical medication (e.g., gel, cream, or ointment)	7.66	6.95	8.17	9.86	0.183
Nasal spray	5.07	4.48	5.57	6.60	0.197
Inhaler-type medication	4.87	3.48	6.43	6.21	0.000
Patch	3.88	2.80	5.02	5.32	0.000
Self-administered injection	2.28	1.16	3.25	4.95	0.000
Injection by a health-care provider	2.67	1.87	3.26	5.14	0.000
Number of Respondents	1000	523	404	73	

Those counted as having a diagnosed condition are those who said yes in Question 8 when asked if they have “ever been diagnosed by a healthcare provider with a swallowing condition”. “Difficulty Swallowing” is defined as having said yes to having difficulty swallowing medications in Q2, and/or having a score of a 3 or more on the EAT-10. Three respondents who did not fit this definition of having difficulty swallowing but had been diagnosed with a swallowing condition are included under those diagnosed with a swallowing condition. The *t*-tests performed are two-sampled, two-tailed tests with unequal variances.

**Table 8:** Logistic regression predicting dysphagia diagnosis.

	Odds Ratio	Std. Err.	*p*-Value
Age (continuous)	0.824	0.031	0.000
Male	0.940	0.093	0.528
Country (comparison group = UK)			
Brazil	1.389	0.244	0.061
China	6.514	0.991	0.000
Indonesia	1.633	0.272	0.003
EAT-10 Score	1.108	0.005	0.000
Constant	0.050	0.009	0.000
Number of Observations	4000		
Log likelihood	−1358.2228		
Prob > chi^2^	0		
Pseudo R Squared	0.268		

## Data Availability

The data have not been deposited in deposited in a publicly available repository but can be made available upon request.

## Supplementary Materials

The following supporting information can be downloaded at: https://www.mdpi.com/article/10.3390/healthcare12181827/s1, Questionnaire (English).
