# Supplementary material for: Dysphagia Prevalence in Brazil, UK, China, and Indonesia and Dysphagic Patient Preferences
Source: Healthcare (Basel). 2024 Sep 12;12(18):1827. doi: 10.3390/healthcare12181827 (PMC11431452; doi:10.3390/healthcare12181827)
Supplement: Supplementary file 1 [file healthcare-12-01827-s001.zip › healthcare-3178454-supplementary.pdf]

# SWALLOWING SURVEY

**[PROGRAMMER NOTES IN BOLD CAPS AND BRACKETS]**

**[DO NOT ALLOW ROUTED SURVEY TRAFFIC]**

**[PROGRAMMER: DISABLE RESUME LATER BUTTON FOR ENTIRE SURVEY]**

**[PROGRAMMER: DISABLE BACK BUTTON FOR ENTIRE SURVEY]**

**[EACH QUESTION APPEARS ON INDIVIDUAL PAGE]**

**[DIGITAL FINGERPRINTING SHOULD BE USED TO AVOID REPEAT PARTICIPATION]**

**INTRODUCTION**

Thank you for your willingness to participate in our survey. This is a brief survey which should take no more than 10 minutes of your time.  The responses you give to our questions are very important to us. Your answers will be kept anonymous and will only be used in the aggregate. If you don't know an answer to a question or if you don't have an opinion, please indicate this in your response. Please do not guess.

Simply click on the ">" button at the bottom of the page to begin the survey.

S1. Please verify that you are human.

**[INSERT Re-CAPTCHA CENTERED ON PAGE]**

S2. In which country do you currently live?

**[INSERT DROP DOWN BOX WITH LIST OF COUNTIRES]**

**[RESPONDENT MUST LIVE IN BRAZIL, INDONESIA, CHINA, OR THE UNITED KINGDOM TO CONTINUE.]**

S3. Are you…?

1. Male
2. Female

**[TERMINATE IF GENDER DOES NOT MATCH PANEL DATA.]**

S4. Please select your age.

**[INSERT DROP DOWN BOX WITH AGES UP TO 99 AND A "Prefer not to answer" OPTION. TERMINATE IF UNDER 18. TERMINATE IF PREFER NOT TO ANSWER.] [TERMINATE IF AGE DOES NOT MATCH PANEL DATA.]**

**[PROGRAMMER: INCLUDE HIDDEN VARIABLE CAPTURING AGE CATEGORY: 18-34, 35-44, 45-54, 55-64, 65+.]**

**[IF COUNTRY = CHINA, ASK S4_A. DISPLAY ON SAME PAGE AS S4.]**

S4_a. Did you report your age using the Gregorian calendar or the lunar calendar?

1. Gregorian calendar
2. Lunar calendar
3. Don't know

**[IF COUNTRY=BRAZIL, ASK S5]**

S5. In which state do you currently live?
**[INSERT DROP DOWN BOX WITH LIST OF 26 ESTADOS/STATES. SEE EXCEL FILE PROVIDED.]**

**[IF COUNTRY=INDONESIA, ASK S6]**

S6. In which province do you currently live?
**[INSERT DROP DOWN BOX WITH LIST OF 34 PROVINCES. SEE EXCEL FILE PROVIDED.]**

**[IF COUNTRY=CHINA, ASK S7]**

S7. In which provincial region do you currently live?
**[INSERT DROP DOWN BOX WITH LIST OF 34 PROVINCIAL-LEVEL ADMINISTRATIVE UNITS. SEE EXCEL FILE PROVIDED.]**

**[IF COUNTRY=UK, ASK S8]**

S8. In which of these countries do you currently live?

1. England
2. Scotland
3. Wales
4. Northern Ireland
5. Other **[TERMINATE]**

S9. This question is a little different. For quality control purposes, please select the number four from the list below.

1. 1
2. 2
3. 3
4. 4
5. 5
6. 6
7. 7

## MAIN SURVEY

### INTRODUCTION

In this survey, we are going to ask about your experience with swallowing, including eating, drinking, and taking medications.

**[NEW SCREEN]**

**[MODULE 1: Measuring Dysphagia via the EAT-10]**

Q1. Some people have problems swallowing, while others do not. To what extent are the following scenarios problematic for you?

Please rate how problematic each scenario is, with 0 meaning "no problem" and 4 meaning it is a "severe problem."

| | 0=No problem 4=Severe problem | | | | |
|---|---|---|---|---|---|
| | 0 | 1 | 2 | 3 | 4 |
| My swallowing problem has caused me to lose weight. | | | | | |
| My swallowing problem interferes with my ability to go out for meals. | | | | | |
| Swallowing liquids takes extra effort. | | | | | |
| Swallowing solids takes extra effort. | | | | | |
| Swallowing pills takes extra effort. | | | | | |
| Swallowing is painful. | | | | | |
| The pleasure of eating is affected by my swallowing. | | | | | |
| When I swallow food sticks in my throat. | | | | | |
| I cough when I eat. | | | | | |
| Swallowing is stressful. | | | | | |

Q2. Do you have difficulty swallowing medications?

1. Yes
2. No **[GO TO Q7]**
3. Don't know/don't recall **[GO TO Q7]**

Q3. How often do you have problems taking a medication as instructed because of swallowing difficulty?

**[ROTATE LIST 1-5 AND 5-1]**

1. Every time I take medication
2. Most times I take medication, but not every time
3. Sometimes, but not most of the time
4. Seldom or rarely
5. Never
6. Don't know

Q4. On a scale of 0 to 4, where 0=No problem and 4 =Severe problem, how problematic is your **difficulty swallowing medications**?

| 0=No problem 4=Severe problem | | | | |
|---|---|---|---|---|
| 0 | 1 | 2 | 3 | 4 |
| | | | | |

Q5. Does your difficulty swallowing medication affect your ability to follow instructions for how often or how long you should take your medication?

1. Yes
2. No
3. Don't know

Q6. Have you ever done any of the following due to your difficulty swallowing medication?

*Select all that apply*

**[RANDOMIZE LIST]**

1. Not fill prescription at all
2. Quit taking a medication
3. Seek alternative treatments that do not include medication
4. Take medication less frequently than the recommended schedule
5. Take fewer pills or tablets at a time than recommended

**[IF RESPONDENT SELECTS SCORE OF 1, 2, 3, OR 4 FOR ANY SCENARIO IN Q1 OR Q4, ASK Q7]**

Q7. Have you ever discussed swallowing issues with a doctor or other healthcare provider?

1. Yes
2. No
3. Don't know/don't recall

Q8. Have you ever been diagnosed by a healthcare provider with a swallowing condition?

1. Yes **[ASK Q8a]**
2. No **[GO TO Q9]**
3. Don't know/don't recall **[GO TO Q9]**

**[IF Q8=1, ASK Q8a. OTHERWISE, GO TO Q9]**

Q8a. If you know, were you diagnosed with dysphagia, odynophagia, or something else?

1. Dysphagia
2. Odynophagia
3. Something else
4. Don't know/don't recall

**[IF RESPONDENT SELECTS SCORE OF 1, 2, 3, OR 4 FOR ANY SCENARIO IN Q1 OR Q4, ASK Q9; IF Q8=1, ASK Q9; OTHERWISE, GO TO NEXT SECTION]**

Q9. Swallowing problems are sometimes experienced by people with certain health conditions, such as head-and-neck cancers, neurological disorders (such as Parkinson's disease or multiple sclerosis), stroke-related impairments, or esophageal conditions. To the best of your knowledge, are your swallowing problems due to another health condition from which you suffer?

1. Yes
2. No
3. Don't know/don't recall

**[MODULE 2: UK ONLY Patient Preferences]**

**[DISPLAY THIS MODULE ONLY IF RESPONDENTS ARE IN THE UK]**

Q10. Do you currently take any prescription medications on a regular basis, for example, daily, weekly, or monthly?

1. Yes
2. No
3. Don't know/don't recall

Q11. For each of the following forms of medication, please indicate whether you have **ever taken** medicine this way in your adult life?

**[RANDOMIZE ORDER]**

1. Oral medication, such as pills, tablets, or liquids that you swallow
2. Suppositories that are inserted anally or vaginally
3. Topical medication, such creams, ointments, or patches
4. Medications that are inhaled using nebulizers, inhalers, or atomizers
5. Medications that are administered by implant
6. Injections, either self-administered or administered by a health-care provider
7. Nasal sprays

**[IF Q11=1, ASK Q12]**

Q12. Thinking about the following types of medication, which of the following have you **ever taken** in your adult life?

**[RANDOMIZE ORDER]**

1. Pills or tablets in pressed powder form that must be swallowed
2. Capsules (active part of the medicine is contained inside a plastic shell)
3. Liquid
4. Tablets that dissolve in your mouth

Q13. Thinking about the medication that you take on a regular basis (i.e., daily, weekly, or monthly), which of the following forms of medication are you **currently taking**?

**[RANDOMIZE ORDER]**

1. Pills or tablets in pressed powder form that must be swallowed
2. Capsules (active part of the medicine is contained inside a plastic shell)
3. Liquid
4. Tablets that dissolve in your mouth
5. Self-administered injection
6. Injection by a health-care provider

7. Inhaler-type medication
8. Nasal spray
9. Patch
10. Topical medication (e.g., gel, cream, or ointment)

Q14. Imagine you had the choice to select the form in which your medication was administered. Please allocate 100 points across the medication formulations below according to your preference. Allocate more points to those formulations which you prefer and less points to those you do not prefer. You may allocate zero (0) points if you do not prefer that formulation.

1. Pills or tablets in pressed powder form that must be swallowed
2. Capsules (active part of the medicine is contained inside a plastic shell)
3. Liquid
4. Tablets that dissolve in your mouth
5. Self-administered injection
6. Injection by a health-care provider
7. Inhaler-type medication
8. Nasal spray
9. Patch
10. Topical medication (e.g., gel, cream, or ointment)

Q15. Which of the following, if any, have you ever tried in attempt to make swallowing of medications easier?

**[RANDOMIZE ORDER]**

| | **Yes** | **No** | **Don't know/Not sure** |
|---|---|---|---|
| Limit the taking of pills to only once a day or every few days | | | |
| Adjusting of posture or the positioning of your head/neck to make swallowing medications easier | | | |
| Learning medication swallowing techniques from a health care provider | | | |
| Learning exercises from a healthcare provider to help build-up swallowing muscles | | | |
| Combining pills or capsule contents with food to make them easier to swallow (e.g., yogurt, applesauce) | | | |

**[MODULE 3: Demographic Questions]**

We are almost finished. Just a few more questions.

Q100. Do you currently suffer from any chronic health conditions for which you take daily medication?

1. Yes
2. No

Q101. Do you or have you ever experienced any of the following health conditions?

1. ALS/Lou Gehrig's disease
2. Arthritis
3. Cleft lip/palette
4. A communication disorder
5. Esophagitis
6. Fibromyalgia
7. Gastro-oesophageal reflux disease (GORD/GERD)
8. Lower back pain
9. Migraines
10. Multiple Sclerosis
11. Myasthenia gravis
12. Neuropathy
13. Parkinson's Disease
14. Pneumonia
15. Stroke
16. Other chronic pain syndrome not listed above
17. None of the above

**[IF COUNTRY=INDONESIA, ASK D_I SERIES]**

D1_I. What is your highest level of education completed?

1. Never/not yet completed primary school
2. Primary school
3. Junior high school
4. Senior high school
5. Vocational high school
6. Diploma I/II
7. Diploma III/Academy
8. Diploma IV/Undergraduate
9. Postgraduate

D2_I. What is your citizenship?

1. Indonesian
2. Foreigner

**[IF D2_I=INDONESIAN, ASK D3_I]**

D3_I. What is your ethnicity?

1. Batak
2. Betawi
3. Javanese
4. Madurese
5. Malay
6. Minangkabau
7. Sudanese
8. Sulawesi
9. Other
10. Don't know

**[IF COUNTRY=BRAZIL, ASK D_B SERIES]**

D1_B. What was the highest level course you have attended?

1. Day care, pre-school (kindergarten and maternal), literacy classes
2. Youth and adult literacy
3. Former primary education (elementary)
4. Former lower secondary education (*ginÃ¡sio* or middle 1st cycle)
5. From 1st to 3rd grade / from 1st to 4th year
6. From 4th to 5th year
7. From 5th to 8th grade / from 6th to 9th year
8. Supplementary elementary school or 1st level
9. Former upper secondary education (scientific, classical, etc., or middle 2nd cycle)
10. Youth and adult upper secondary education
11. College
12. Graduate specialization (at least 360 hours)
13. Master
14. Doctorate

D2_B. Your color or race is:

1. White
2. Black
3. Yellow
4. Brown
5. Indigenous

**[IF COUNTRY=CHINA, ASK D_C SERIES]**

D1_C. What is the highest level of education you have completed?

1. Never been to school
2. Basic literacy class
3. At least some primary school
4. At least some junior high school
5. At least some senior high school
6. At least some secondary training school
7. Some college
8. University degree
9. Graduate degree

D2_C. Please indicate your ethnicity.

1. Han
2. Minority

**[IF COUNTRY=UK, ASK D_U SERIES]**

D1_U. What is the highest level of education you have completed?

1. Primary school
2. Secondary school up to 16 years
3. Higher or secondary or further education (A-levels, BTEC, etc.)
4. College or university
5. Post-graduate degree

D2_U. What is your ethnic background?

1. White
2. Asian or Asian British
3. Black, Black British, Caribbean or African
4. Mixed or Multiple ethnic groups
5. Other ethnic group

**Thank you. Those are all of the questions we have for you today.**
